# Who chooses prepaid dental care? A baseline report of a prospective observational study

**DOI:** 10.1186/1472-6831-14-146

**Published:** 2014-12-03

**Authors:** Charlotte Andrén Andås, Magnus Hakeberg

**Affiliations:** Department of Behavioral and Community Dentistry, Institute of Odontology, Sahlgrenska Academy, University of Gothenburg, Göteborg, Sweden

**Keywords:** Capitation, Fee for service, Dental insurance, Oral health, Lifestyle

## Abstract

**Background:**

An optional capitation prepayment system has been implemented in Swedish dental care, supplementary to the traditional fee-for-service scheme within the Public Dental Service. The implementation of a new system may have a variety of preferred and adverse effects, arguably dependent on the individual patient’s attitudes, health beliefs and course of action.

The aim of this study was to describe potential differences regarding socioeconomic and lifestyle factors, perceived oral health and attitudes towards oral health between patients in the two payment systems.

**Methods:**

Questionnaire data were consecutively collected from 13,719 patients, who regularly attended 20 strategically selected clinics within the PDS in Region Västra Götaland, before they were offered the choice between the traditional and the new payment system.

**Results:**

Capitation patients were more often female and well educated. They had healthier habits, were more motivated to follow self-care advice, more often judged their oral health to be very good and considered oral health to be very significant for their wellbeing. The results were statistically significant and described a gradient.

**Conclusions:**

The more explicitly affirmative the answer, the more likely the patient was to choose the prepayment scheme. There appears to be a pattern of differences with respect to important individual views on oral health between patients choosing a capitation system or a fee-for-service system. These differences may be important when assessing outcomes in the new payment system and in public dental care.

## Background

The organization of dental care financing differs widely between geographical areas and between countries [1]. In Scandinavia as a whole, and in Sweden in particular, the average degree of public involvement has been comparatively high over time. In Sweden, a National Dental Insurance scheme that reimbursed all types of dental care treatment was introduced in 1974 [2]. The scheme covered all residents in Sweden and included both private and public dental care suppliers. The aim was to make dental care accessible to all residents on equal terms, thereby realizing a social policy stance with the objective that social class should not be obvious from looking at a person’s teeth [2]. At the start, the coverage was far-reaching, with reimbursements amounting to 50% of dental care costs up to SEK 1000, and up to 75% of fees above that level, based on fixed tariffs that were mandatory to all caregivers. In response to the gradually increasing costs of the insurance, the percentage reimbursed to the patient was gradually reduced. The scheme was reconstructed in 1999, with a lower degree of reimbursement for basic oral health care, irrespective of dental care need.

Dental care in Sweden is provided by the Public Dental Service (PDS), organized by the local county councils, and by private practitioners. The Västra Götaland region, from which the data in the present study were collected, has an adult population of about 1.2 million inhabitants. Approximately 45% of all adults are registered with the PDS. Dental care providers in Sweden have traditionally been reimbursed on a fee-for-service basis. In 1999, the law was changed to allow providers to offer prepaid dental care in the form of a capitation payment system to their adult patients [3]. As a result, the Public Dental Service in a few county councils introduced their own version of a capitation payment scheme, which initially gained some ground [4]; however, after ten years, the scheme only covered about 5% of the adult population. In 2010, a uniform capitation payment scheme was implemented nationwide by all 21 Swedish Public Dental Service organizations in the respective county councils. This uniform capitation payment system involves signing a three-year contract with the caregiver to pay a risk-differentiated fee and to maintain an individually designed self-care plan in exchange for receiving all necessary basic dental care; a model corresponding to a dental insurance policy. As of 2014, 25–30% of all adult patients receiving dental care from the PDS in Region Västra Götaland are included in the capitation system.

The introduction of a supplementary optional insurance-like payment system in Swedish dental care corresponded to the development of reimbursement models within the health care system, aimed at satisfying the simultaneous requirements of cost containment and quality improvement [5, 6]. It could be assumed that the introduction of the capitation payment system would lead to changing incentives for patients as well as caregivers, thereby altering the volume both of requested and provided dental care in general, and preventive dental care in particular [7, 8]. Furthermore, access to a new optional payment system may encourage the patients to make their own (informed) decisions, and may also contribute to advancing patient participation and empowerment with regard to oral health. From an economic theory perspective, it would be expected that those who choose the capitation payment system rather than the fee-for-service system; i.e., those who choose a voluntary health insurance policy, would be those with a higher-than-average risk of needing dental care [9]. However, empirical studies of the demand for voluntary health insurance generally fail to find support for such a relationship [10–13]. On the contrary, insured individuals tend to be healthier, younger and better educated than those who are uninsured [14, 15].

The choice of health care insurance was traditionally described according to the maximum utility model [9], and was thereby considered to be a function of the expected expenditure and care demand. Later, it has become increasingly obvious that factors other than these strictly theoretical assumptions also influence the decision whether or not to buy health insurance; factors such as those involved in making individual rational decisions based on adequate information. Thus, additional explanatory variables, such as age, sex, level of education, anticipated future health, and satisfaction with the present care provider [16], have become increasingly important, in addition to the strictly economic variables of price, deductibles and out-of-pocket payments. Reports on the influence of variables related to individual characteristics on the choice of insurance are, however, sparse and, when available, most often apply to the US market.

The results from a randomized study, one of very few looking into dental insurance schemes, indicated that a more generous insurance coverage was correlated with improved oral health in patients younger than 35 years of age, and especially in those with the poorest oral health [17]. In agreement with an ambition of equity in health, it may be of societal importance to discover any adjustable health inequalities that might result from the introduction of a new payment system [18].

Consequently, several possible scenarios may follow from the changes to the payment systems in Swedish dental care, where the individual patient’s opinion and course of action may be considered to be highly influential. The aim of this study was to describe differences with regard to socioeconomic and lifestyle factors, as well as perceived oral health and attitudes towards oral health among patients choosing a capitation prepayment system or a fee-for-service payment system for dental care within the Public Dental Service in Region Västra Götaland, Sweden.

## Methods

### Subjects

Data were collected from 13,719 patients who were consecutively enrolled when attending their scheduled regular appointments for a dental examination, if they attended one of 20 PDS clinics selected through a stratified, random procedure. The procedure randomly sampled 20 clinics out of a total of 116 clinics in Region Västra Götaland, stratified by urban/rural area and by administrative regional subdivision. The region covers about 1.2 million persons over the age of 20, 40–45% of whom are registered with the PDS. The remaining adult individuals receive their dental care in the private dental care sector.

The inclusion criteria were: age 20 years and older, accepting to participate by filling in a questionnaire, and ability to read and understand Swedish. The study was initiated on May 1^st^, 2007, during the regional implementation of the new optional prepayment scheme Frisktandvård (‘Dental Care for Health’), and inclusion continued during 2007 and 2008. A questionnaire was filled out before the scheduled dental examination, and before the patient had the opportunity to choose between the new and the traditional payment systems. After the dental examination, where clinical data were recorded, each patient was informed about the respective payment systems and asked to choose between them. Thus, the data collection may be considered to constitute a natural experiment - the implementation of an elective transition of payment system in Region Västra Götaland, Sweden.

### Risk classification

After the clinical examination and completing the questionnaire, the individual patient was assigned to one of five risk groups. The classification was computer-assisted by software linked to the electronic patient chart system T4 (T4 Practice Management software, Carestream Dental, Stockholm, Sweden). A proposed risk group classification was established in a systematic way: Status information on caries, periodontitis, previously received care and the presence/absence of wisdom teeth was automatically transferred from the individual patient chart into the risk classification software. Manually entered modifying factors included medication, if any, tobacco use, levels of oral hygiene and tooth wear. All factors were marked on a three-grade scale and weighted using a defined algorithm into a proposed risk classification that could be altered in either direction by the responsible dentist/dental hygienist. Each risk group was linked to a fixed fee, which represented the cost for the patient to consider if they wanted to make an active choice and change from the fee-for-service payment system to the prepayment agreement.

### The agreement

If, and when, the patient agreed to pay the fee stipulated for the risk group in question, he/she entered an agreement, or contract, with the dentist/dental hygienist with certain obligations for both parties: The dentist/hygienist would invite the patient to (mandatory) regular dental examinations every 12–18 months, depending on risk group, and agree to provide all dental care needed during the following three-year period. Specialist treatment and fixed prosthodontics were not included. The patient also had to commit to an individually designed self-care protocol, including advice concerning oral hygiene, diet and fluoride usage. When the three-year period expired, a renewed risk classification was performed, and the patient was again offered the possibility to choose between the prepayment scheme, at a fee determined by an updated risk classification, or traditional fee-for-service payment, for the next three-year period.

## The method

All data sets included clinically recorded measures of oral health, together with a questionnaire focusing on health and health beliefs. Patients were followed at 12 or 18-month intervals, and after 3 and 6 years, respectively, they were again given the opportunity to change or to stick with their present payment scheme; prepayment or fee-for-service payment.

The questionnaire obtained information about demographics, self-reported oral and general health, lifestyle, dental care habits, preventive measures, experience of dental care, attitudes and beliefs towards health and disease. The procedure was standardized through formalized instructions to the dental staff to ensure that the questionnaire was completed by the patient him/herself before the clinical examination. All completed questionnaires were stored at the respective clinics until the data collection was completed and the data were transferred to a computer file. The Regional Ethical Review Board in Gothenburg has approved the study (No. 323–07).

### Included variables

The dependent variable indicated the patient’s choice of either of the two payment schemes:

- 0 = The traditional fee-for-service scheme
- 1 = The prepayment scheme; i.e., the new dental insurance policy Frisktandvård (‘Dental care for health’).

The independent variables included the answers to the following questions in the questionnaire.

The response options to some questions were trichotomized for the multiple regression analysis merging low value options, as described in Table 1.

**Table 1:** **Items in the questionnaire and respective response options**

Question	Label	Response options after collapsing
*“When were you born?”*	yymmdd	
*“Please indicate your gender”*		0 Female
		1 Male
*“How tall are you?”*	cm	
*“How much do you weigh?”*	kg	
*“What is your highest completed level of education?”*		1 Elementary School, not finished +
		Elementary School, finished 2 Upper Secondary School
		3 University
*“How do you assess your own dental health at present?”*		1 Bad +
		Somewhat bad
		2 Good
		3 Very good
*“Do you smoke?”*		1 Yes +
		No, but used to
		2 No
*“How much do you exercise in your spare time?”*		1 Almost no exercise at all +
		Short walks, now and then
		2 Regularly, once a week +
		Regularly, twice or more a week
		3 Regularly, hard, at least twice a week
*“Have you been motivated to follow advice and instructions that you have received concerning your oral health?”*		1 No +
		Yes, a bit motivated
		2 Yes, quite motivated
		3 Yes, very motivated
*“In your opinion, do your dietary habits affect your oral health?”*		1 No +
		Yes, a little
		2 Yes, somewhat
		3 Yes, very much
*“In your opinion, how significant is good oral health for your general wellbeing?”*		1 Not at all significant +
		A little
		2 Somewhat significant
		3 Very significant
*“How satisfied are you with the appearance of your teeth?”*		1 Very dissatisfied +
		Quite dissatisfied
		2 Quite satisfied
		3 Very satisfied

### Data analysis

Analyses were performed using the SPSS, version 20.0. The Mann–Whitney U test and the chi-square analysis were used to detect statistically significant differences in the distribution of questionnaire responses between the two payment schemes, for continuous and ordinal-scale independent variables, respectively.

A logistic regression model was developed using a stepwise forward strategy. Independent variables were categorized as covariates or confounders, according to our graphically outlined understanding of their relationship. All the available independent variables were considered for the final logistic regression model after they were determined not to be correlating hazardously with each other, based on the correlation analysis (Spearman’s ρ ≤ 0.348) or when cross-tabulated, variable by variable. Each independent variable was then independently included in the final regression model if it exerted a statistically significant influence on the value of the dependent variable in the bivariable analysis (p < 0.25 in the log likelihood test), together with a similarly statistically significant influence in the multivariable analysis (p < 0.25 in the Wald test). A number of interaction terms; for instance, the product of the variables *age* and *assessment of own oral health* were considered but rejected, as they failed to show statistical significance as described above. The final full model aimed at predicting the choice of payment scheme and to explain the amount of variability assessed by the independent variables. Results were expressed as odds ratios (OR) with 95% confidence intervals. The traditional payment scheme was used as the reference category of the dependent variable *payment system*.

## Results

Table 2 presents the distribution of age, BMI and gender between the two payment models, and how the answers to the questions in the questionnaire varied between the different response options for the two payment models, respectively. The patients who chose to prepay differed statistically significantly from those who chose to pay traditionally; for instance, by being younger (mean age 34.9 yrs and 43.1 yrs, respectively), and more often female (56% and 51%, respectively).

**Table 2:** **Distribution of answers from the questionnaire, in total and as comparisons between the two payment schemes**

	Total				Prepayment scheme				Traditional scheme				***p***
	Mean	SD	N	%	Mean	SD	N	%	Mean	SD	N	%	
**Age**	40.34	12.33			34.90	9.94			43.07	12.10			<0.001
**BMI**	24.84	3.87			24.30	3.72			25.14	3.92			<0.001
**Sex**													
Female			7225	52.7			1783	55.9			4608	51.3	<0.001
Male			6495	47.3			1409	44.1			4366	48.7	
**Education**													
Elementary School			1558	11.5			165	5.2			1241	14.0	<0.001
Upper Sec. School			7560	55.8			1887	59.7			4801	54.2	
University			4432	32.7			1109	35.1			2810	31.7	
**Assessment of own oral health**													
Bad/ Somewhat bad			4250	31.3			672	21.2			3047	34.3	<0.001
Quite good			7437	54.7			1873	59.3			4774	53.7	
Very good			1900	14.0			616	19.5			1068	12.0	
**Smoking**													
Yes			1507	11.1			233	7.3			1037	11.7	<0.001
No, but used to			2361	17.4			468	14.8			1635	18.4	
No			9739	71.6			2471	77.9			6225	70.0	
**Spare time exercise**													
No/Short walks			4479	33.2			863	27.3			3086	34.8	<0.001
Regularly 1-2/week			6934	51.1			1672	52.9			4554	51.3	
Regularly, hard, 2/week or more			2150	15.8			630	19.9			1231	13.9	
**Motivation to follow self-care instructions**													
No/ Yes, a little motivated			2692	21.8			552	17.4			2064	23.5	<0.001
Yes, quite motivated			6005	44.1			1420	44.9			3926	44.1	
Yes, very motivated			4637	34.1			1192	37.7			2905	32.7	
**Thinking dietary habits affect oral health**													
No/A little			1803	13.3			312	9.8			1254	14.1	<0.001
Yes, some			4861	35.8			1037	32.7			3310	37.3	
Yes, very much			6932	51.0			1820	57.4			4319	48.6	
**Significance of oral health for well-being**													
No/ Little significance			516	3.8			81	2.6			352	3.9	<0.001
Some significance			5647	41.6			1296	40.1			3741	42.1	
Very big significance			7424	54.6			1818	57.4			4783	53.9	
**Satisfaction with teeth’s appearance**													
Very/Quite dissatisfied			2198	16.2			376	11.8			1538	17.4	<0.001
Quite satisfied			9800	72.1			2324	73.4			6432	72.4	
Very satisfied			1596	11.7			468	14.8			911	10.3	

There were statistically significant differences in the distribution of responses between the prepayment scheme and the traditional payment scheme for all questions. Moreover, the prepayment group answers displayed a higher level of education, better self-assessed level of oral health, a lower incidence of present and former smoking, higher levels of spare time exercise and greater motivation to follow self-care advice. They also revealed a stronger belief in dietary habits affecting oral health as well as in oral health being significant for general wellbeing. The prepayment group also reported greater satisfaction with the appearance of their teeth. All differences were statistically significant.

The results from the bivariate and the multivariate logistic regression models are shown in Table 3. The more affirmative the answer to a question in the questionnaire (in the case of smoking, the more affirmative answer being “no”), the more likely the person was to choose the prepayment scheme; i.e., the higher the odds ratio. The gradient of increasing odds ratios was maintained from the bivariate into the multivariate regression model for a majority of the variables, which could be considered to increase the credibility of the association, as all the variables were included in one model. For both the variable of smoking and the variable of the significance of oral health for general wellbeing, the multivariate model showed increased odds ratios compared with the bivariate model, as opposed to all the other variables, where the effect was diluted when the other variables were controlled for. The Nagelkerke test model evaluator showed a value of 0.17, which can be interpreted as there still being other major variables involved in explaining and predicting the patients’ choice of payment system in dental care.

**Table 3:** **Logistic regression model of variables influencing the choice of payment scheme**

	Bivariate		Multivariate	
	OR	CI	OR	CI
**Age**	0.94	0.94-0.94	0.94	0.93-0.94
**Sex**	1.0		1.0	
	1.20	1.10-1.30	1.10	1.00-1.21
**Education**				
Elementary	1.0		1.0	
Upper sec. school	2.96	2.50-3.51	1.34	1.11-1.62
University	2.97	2.49-3.54	1.50	1.24-1.82
**Smoking**				
Yes	1.0		1.0	
Yes, former	1.27	1.07-1.52	1.43	1.18-1.73
No	1.77	1.52-2.05	1.41	1.20-1.67
**Spare time exercise**				
No	1.0		1.0	
Regularly 1-2/w	1.32	1.20-1.45	1.17	1.06-1.30
Regularly >2/w	1.83	1.62-2.07	1.17	1.02-1.34
**Assessment of own oral health**				
Bad/Not good	1.0		1.0	
Good	1.78	1.61-1.97	1.54	1.38-1.71
Very good	2.62	2.30-2.98	1.87	1.62-2.16
**Motivation to follow self-care instructions**				
None/Little	1.0		1.0	
Quite motivated	1.35	1.21-1.51	1.30	1.15-1.47
Very motivated	1.53	1.37-1.72	1.49	1.30-1.70
**Significance of oral health for wellbeing**				
None/Little	1.0		1.0	
Somewhat	1.47	1.15-1.89	1.53	1.17-2.00
Very significant	1.65	1.29-2.12	1.72	1.31-2.26

Nagelkerke 0.17.

## Discussion

This study of responses from a questionnaire completed by 13,719 regularly attending PDS patients in Region Västra Götaland, Sweden, reported on individual preferences regarding individual health investments and the value of good oral health. The responses differed significantly between those who chose the fee-for-service system and those who chose the new optional prepayment system Frisktandvård (‘Dental care for health’). The study further showed a gradient regarding the influence of the investigated aspects: lifestyle, socioeconomic factors, assessment of own oral health, and the significance of oral health for general wellbeing, on the patient’s choice of payment system. The more explicitly affirmative response option chosen by the patient, the more likely she/he was to choose the prepayment scheme, whether the variable was analyzed separately or the influence of all other variables in the study were controlled for.

Earlier studies on capitation payment in Swedish dental care have indicated differences in the patients’ general health between payment systems [19], as well as an association between the payment system and oral health-related quality of life [20], where capitation system patients showed both better self-reported general health and higher oral health-related quality of life than fee-for-service patients. This appears to be in accordance with the results of the present study with regard to variables representing assessment of own oral health and the significance of oral health for wellbeing.

The results from this study further agree with previous empirical studies on voluntary health insurance [14, 15], thus disputing standard economic theory, which assumes that the higher-than-average risk patients are more inclined to choose an optional health insurance. A rationale for this presumption may be that a minor expected benefit from taking out an insurance for individuals with relatively low risk would make them more likely to refrain from doing so than individuals with a relatively high risk, with a relatively greater expected benefit. Therefore, a positive correlation could be expected between health risk and voluntary health insurance, and – since health risk may be assumed to be negatively dependent on health – a negative correlation between health and insurance. However, recent empirical evidence apparently points in the opposite direction. This has generally been explained in the literature by correlating the individual risk with risk preference [15, 21], meaning that individuals with better health may be more averse to risk than individuals with poorer health. The rationale would thus be that the more averse to risk an individual becomes, the more inclined he or she will be to make health investments that reduce the probability of ending up with bad health. This seems to mirror the results of the present study, as patients with better self-reported oral health and a better lifestyle significantly more often chose the prepayment system.

The diversification of payment systems in Swedish dental care introduced new incentives for caregivers as well as for patients [8, 22]; for example, payment per item of dental care (fee-for-service payment) offers an incentive for the caregiver to match the magnitude of treatment proposed to the patient to his/her available time, so called supplier-induced demand. The risk of overtreatment due to supplier-induced demand may be further amplified by the presence of a third party financier by reducing the patients’ costs for dental care procedures through a high-cost protection scheme. Thus, fee-for-service payment holds no incentive to constrain the amount of treatment [23, 24]. When, on the other hand, caregivers receive a fixed amount for each patient, regardless of the patient’s individual care need, as in the capitation scheme, they may feel motivated to minimize the time spent on each patient. Capitation payment may thus involve a risk of undertreatment, rather than overtreatment (supplier-induced demand) [7]. In a longer perspective, however, the incentive for caregivers as well as for patients to keep costs down could instead, in theory, strengthen the motivation of both parties to take preventive action. To attract the interest of caregivers in investing in time-consuming preventive treatment they need to perceive a decent prospect of gain from their investment. This, in turn, requires that the caregiver-patient relationship be maintained over a longer period of time, as is possible when linked by a contract [6]. The potential gain from clearly targeting preventive oral health care could be either continuously improved oral health for the individual patient [25], or reduced costs for the patient as well as for society, as some forms of treatment will no longer be necessary. This reasoning received some support in a review article on the care content in capitation versus fee-for-service payment systems [8], which indicated a difference between the payment systems with more preventive care received by capitation patients, and more restorative treatment received by fee-for-service patients. Ultimately, it could also be argued that the higher degree of education and health awareness shown by the capitation system patients may further increase the difference in oral health development between patients in the two different payment systems over time. Theoretical reasoning and empirical findings along these lines have been demonstrated by Marmot, with regard to general health [18].

The strengths of this study may be considered to be the large number of participants from 20 systematically selected clinics, and the fact that data were collected before the first possible opportunity to choose between a traditional payment system and a new system with considerably different characteristics. Among the study’s weaknesses are those that follow from a natural experiment in its live setting: There is a risk of classification problems due to difficulties of ensuring internal validity in a large number of study centers. Integrated in the quasi-experimental design is also the danger of over-interpreting the results due to selection bias. One reason to suggest an adequate representativity of the sample, apart from the sample size, is the approximate agreement between the genders in the study (male 47.3%, female 52.7%) and the overall gender distribution in the total adult PDS patient population in the Region Västra Götaland (male 48%, female 52%).

## Conclusions

In conclusion, the present study indicates that patients’ self-reported attitudes concerning the impact of good oral health differ between those who chose a new insurance-like payment system, and those who chose the traditional fee-for-service system. It may be argued that this difference is linked to variations in the perception and management of risk. The results also suggest a pattern indicating that patients are more likely to choose the prepayment scheme if they are female, well educated, do not smoke, exercise extensively, assess their own oral health as very good, are very motivated to follow self-care advice, and think that oral health is very significant for general wellbeing.

Encouraging patients to initiate a cycle of good oral health is a major challenge in everyday dental health care. In the light of the reasoning around the results of this study, signing up for an optional health insurance may be regarded as influential when it comes to encouraging oral health improvement. The findings may contribute to putting the focus on the potential of extending the new payment system’s eligibility and accessibility further among the heterogeneous group of adult PDS patients.

## Consent

Written informed consent was obtained from the patients for the publication of this report.

## Abbreviations

PDS: Public Dental Service
OR: Odds ratio
SEK: Swedish currency.
